# Virtual reality in managing Complex Regional Pain Syndrome (CRPS): a scoping review

**DOI:** 10.3389/fneur.2023.1069381

**Published:** 2023-09-07

**Authors:** Mauricio Arcos-Holzinger, Johanna Theresia Biebl, Claudia Storz, Marcus Gutmann, Shahnaz Christina Azad, Boris Michael Holzapfel, Eduard Kraft

**Affiliations:** ^1^Department of Orthopaedics and Trauma Surgery, Musculoskeletal University Center Munich (MUM), University Hospital, LMU Munich, Munich, Germany; ^2^Facultad de Medicina, Universidad CES, Medellin, Colombia; ^3^Department of Anaesthesiology, University Hospital, LMU Munich, Munich, Germany; ^4^Interdisciplinary Pain Unit, University Hospital, LMU Munich, Munich, Germany

**Keywords:** virtual reality, Complex Regional Pain Syndrome, immersive reality, chronic pain, body perception disturbances

## Abstract

**Background:**

Complex Regional Pain Syndrome (CRPS) is a severe pain disorder that does not yet have a specific treatment. Patients with CRPS not only suffer from a wide range of symptoms that affect their quality of life but also present psychological affections to the way they see their body and specifically their affected limb. Virtual Reality (VR) modalities have become a targeted treatment for chronic pain and in the case of CRPS, may be a valuable approach to the mechanisms that affect these patients.

**Objectives:**

Using the PRISMA Scoping Review guidelines, we intend to uncover the key information from the studies available about VR modalities in the treatment of CRPS. We focus on the improvement of pain levels, body perception disturbances (BPD), and limb movement/daily function.

**Results:**

Our search strategy resulted in 217 articles from PubMed. Twenty were assessed for eligibility and seven were included in the final qualitative synthesis. Of these seven articles, we included a clinical trial, three pilot studies, a blinded randomized controlled trial, a crossover double-blind trial, and a randomized controlled trial. These studies provide important subjective patient findings, along with some statistically significant results in the experiences of VR therapies modulating pain, BPD, and improving limb movement/daily function. However, not all the studies included statistical analysis, and there are contradicting data found from some patients that did not perceive any improvement from VR therapies.

**Conclusions:**

We describe the results found in 7 articles that focus on the treatment of CRPS with VR modalities. Overall, the articles have various limitations, but the strategies related to immersive virtual reality, cardiac signaling, body switching and limb modulation have shown the most promising results for pain reduction and BPD improvement. These strategies reflect on pathophysiological mechanisms that are hypothesized to be affected in CRPS patients leading to the chronic pain and BPD that they experience. Not much evidence was found for improvement in limb movement and daily function. This review is a pathway for future studies on this topic and a more extensive data synthesis when more information is available.

## 1. Introduction

Complex regional pain syndrome (CRPS) is a severe disorder of chronic pain disproportionate to any inciting event following an injury to a limb. CRPS includes sensory, vasomotor, sudomotor, and/or motor/trophic signs and symptoms, which are included in the Budapest criteria for the diagnosis of CRPS. Examples include allodynia, hyperalgesia, skin color changes, sweating, edema, trophic changes, and motor dysfunction (1).

CRPS can be divided into type 1 and type 2 depending on if there is identifiable damage to a nerve (2). The upper limbs are usually more affected than the lower limbs in adults. The most common events leading to this disorder are fractures (mainly distal radius), contusions/sprains, and surgery. However, 10% of cases do not have an identifiable precipitating event (3).

Epidemiologically, CRPS is more frequent in females than in males, with a ratio of 3.5:1 (4, 5). In a population study in Minnesota, an incidence rate for CRPS type 1 of 5.46 per 100,000 person-years and a period prevalence of 20.57 per 100,000 were calculated. On the other hand, the CRPS type 2 incidence rate was just 0.82 per 100,000 person-years (6).

Regarding the etiology, diagnosis, classification, and treatment of patients with CRPS, various arguments and discordances exist (7), but many authors have hypothesized a pathophysiological explanation for CRPS, which includes classic inflammation, neurogenic inflammation, impairment of the autonomic nervous system, and central nervous system plasticity (6).

Additionally, no high diagnostic value serum markers or imaging findings have been identified, making the diagnosis purely clinical.

Similar to other chronic pain disorders there are also a range of psychological aspects to CRPS (8). Patients may experience distortions to affected limb positions, sizes, and the peripersonal space surrounding the body (9). These disorders found in CRPS patients are described as body perception disturbances (BPD) and are closely related to the disturbances in body scheme (a dynamic, real-time representation of one's own body) and body image (a conscious visual representation of the way the body appears from the outside) (10). Body scheme and body image are two distinct yet interacting concepts (11).

The treatment options available for CRPS are multimodal, involving medications for partial processes (bisphosphonates, glucocorticoids, and NSAIDs), physical therapy, occupational therapy, interventional strategies and psychotherapy, that altogether do not establish a specific treatment for these patients (6, 12). Along with movement exercises, manual therapy techniques and advice on aids, treatment methods such as mirror visual feedback (MVF), neurocognitive rehabilitation developed by Perfetti and the graded motor imagery (GMI) are used in CRPS rehabilitation (13). Additional physical stimulation therapies include electrotherapy, neuromodulating procedures, manual lymphatic drainage, CO2 applications and paraffin wax baths (14). In a study conducted in Korea, it was shown that CRPS patients had low overall satisfaction with the rehabilitation services they received despite multimodal approaches, stressing the need for improved and innovative therapies (15).

Virtual reality (VR) therapies have emerged for the treatment of chronic pain and have shown promising results for various conditions (16, 17). With regard to the application of VR-based therapies in neurorehabilitation, Hao et al. showed in their meta-analysis that changes in neuronal plasticity correlated positively with functional recovery in patients who received such therapy after stroke. They hypothesized the involvement of mirror neurons as one of the possible specific neurological mechanisms of this therapy (18). A recent meta-analysis by Calafiore et al. suggested that in patients suffering from multiple sclerosis (MS), rehabilitation interventions using VR seem to be more effective in improving balance in MS patients than conventional rehabilitation interventions (19).

Virtual reality is characterized by the attempt to replace real sensory impressions with an artificial, computer-generated environment (20). In categorizing the different entities of VR, presence, immersion and interactivity serve as cornerstones of closely interrelated phenomena (21). Immersion corresponds to the user's engagement with a VR system and is referred to by Slater as the objective property of a system. Particular emphasis should be placed on so-called sensory immersion, which according to Kim et al. corresponds to the degree to which the range of sensory channels is addressed by the virtual simulation (22). Presence in the VR context, according to Slater, is the psychological illusion of “being there” even though one knows with certainty that one is not. According to the author, this is a perceptual illusion, but not a cognitive one (23). Interactivity, in turn, is closely related to immersion, in the sense that immersion is a function of the VR hardware that creates the illusion of a physical presence in a non-physical world (24). According to Kilteni et al., embodiment in the VR context describes the sense that our self is in a virtual body, that we control this body, and that this body belongs to us. The term embodiment includes three subcomponents: self-location, agency, and body ownership (25). In addition to distraction, which seems to be particularly relevant in the treatment of acute pain, possible therapeutic effects of VR interventions for chronic pain syndromes include the development of coping skills, facilitation of activities of daily living, positive mood induction and reduction of fear of movement (26). The treatment of BPD is a specific challenge in CRPS patients. In this regard, virtual embodiment and body transformation illusions through VR can be used to address the physiological perception of patients with these disturbances (27). Furthermore, VR-based therapies in CRPS could address the phenomenon described by Di Pietro et al. that the putative reduction in somatosensory representation of the affected limb in CRPS is actually due to an increase in representation of the unaffected limb (28). In addition, Filbrich et al. reported that CRPS patients showed deficits in visuospatial perception (29). It is possible that VR could be used to specifically target these deficits. Moreover, VR rehabilitation engages several cortical and subcortical neuronal circuits that potentiate patients' learning and recovery since cortical reorganization is hypothesized to be involved in sensory and motor impairments of patients with CRPS (30).

In this scoping review, we intend to synthesize the information available about studies analyzing the effectiveness of VR in managing pain, BPD, and daily function/limb movement in patients with CRPS. By this, we hope to impulse the use of VR for CRPS and open the way for future clinical trials, systematic reviews, or meta-analyses (31).

## 2. Methods

### 2.1. Protocol

To conduct this scoping review, the *Preferred Reporting Items for Systematic reviews and Meta-Analyses extension for Scoping Reviews (PRISMA-ScR) Checklist* was followed for the most part (http://www.prisma-statement.org/Extensions/ScopingReviews). The PubMed database was consulted using the search strategy provided below, and the articles found were selected using the eligibility criteria created.

### 2.2. Eligibility criteria

We included studies that evaluated VR-based therapies for adult patients with CRPS.

Studies with pediatric participants and those that were not written in English were excluded.

### 2.3. Search selection

As shown in the PRISMA flowchart and following the established eligibility criteria, we selected the most suitable papers published from 2010 to 2021 that met our objectives. The search strategy was executed on the 26th of November, 2021.

A team of two therapists (CS, MG), two pain specialists (SCA, EK), a neurologist (EK), an orthopedic and trauma surgeon and Fellow of the European Board of Orthopaedics and Traumatology (BMH), a resident in rehabilitation medicine (JTB), and a research associate (5th year medical student) (MA-H) performed the review.

### 2.4. Search strategy

The following search strategy was developed using the Mesh terms found on PubMed that met the eligibility criteria:

((Virtual Reality (MESH) OR Virtual Reality Exposure Therapy (MESH)) AND (CRPS (MESH) OR Causalgia (MESH) OR Complex Regional Pain Syndromes (MESH) OR Reflex Sympathetic Dystrophy (MESH) OR Body Image (MESH)))

## 3. Results

**Figure 1:**
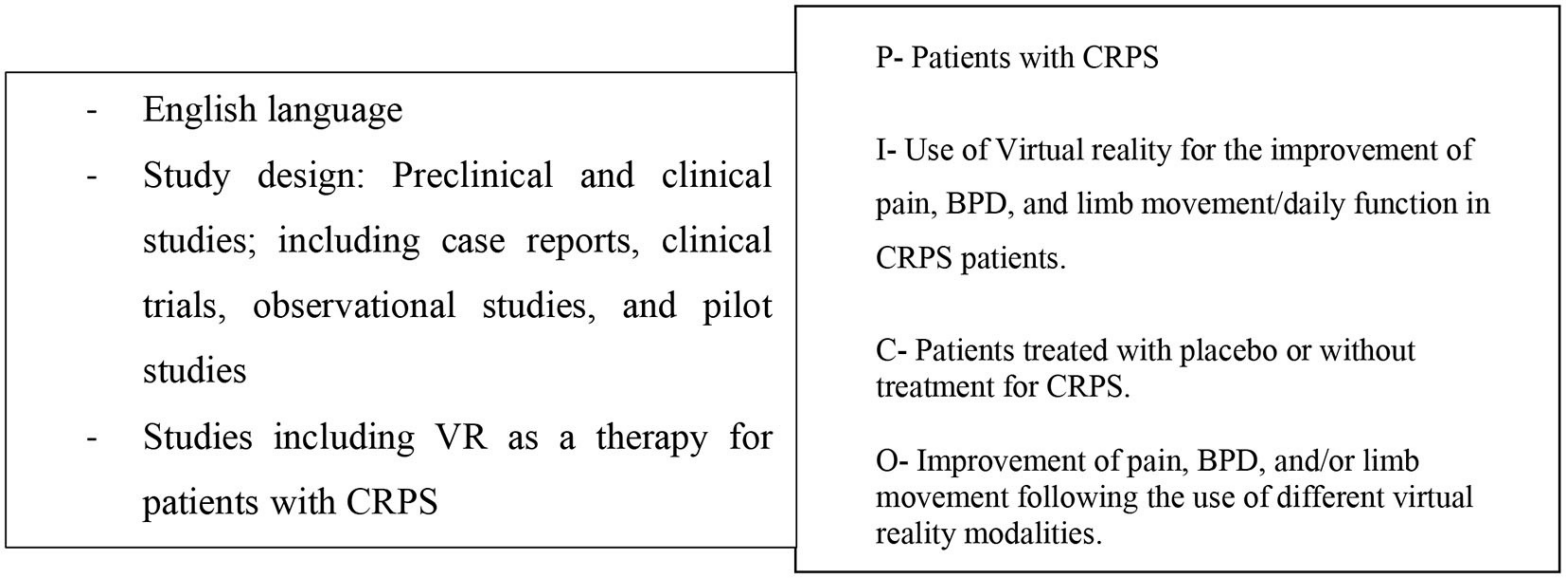
Eligibility criteria and PICO-Model: patient/population, intervention, comparison, outcome.

**Figure 2:**
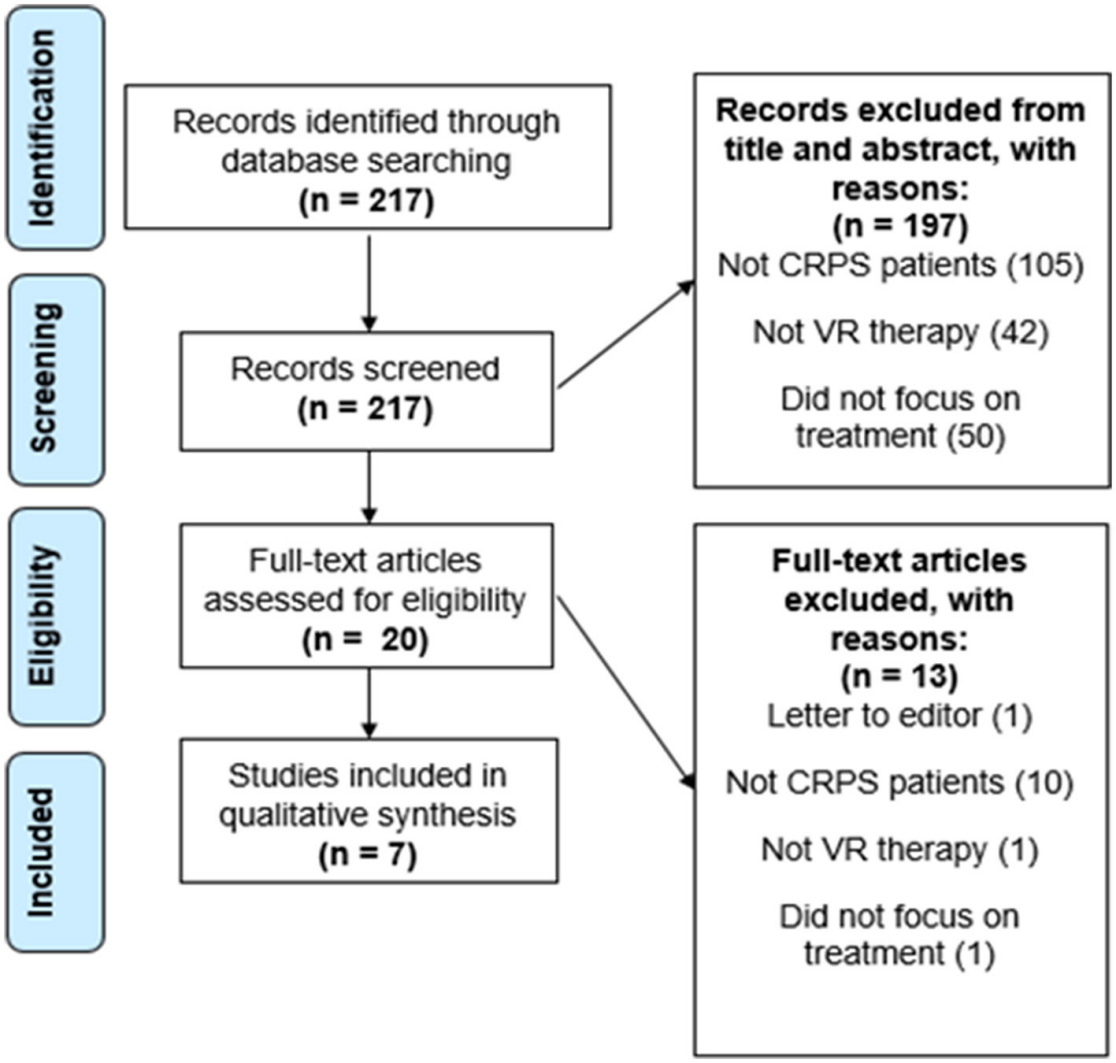
Flow diagram illustrating the search and selection process.

A total of 217 papers resulted from the primary search on the PubMed database. From the 217 papers that resulted, 197 were excluded after revising the title and abstract. These were all excluded for not showing any information in the title and abstract that indicated inclusion of CRPS patients, use of a VR therapy, or did not focus on the treatment of these patients but instead on their diagnosis or prediction, which was not the intended focus of this review. The 20 articles left were assessed for eligibility. This step resulted in the exclusion of 12 articles for not showing CRPS patients, not focusing on VR therapy, or not focusing on the treatment of the patients. One more article was excluded for being a letter to the editor and because it focused on pediatric patients. Finally, our search resulted in a total of 7 papers that were left for the final analysis and qualitative synthesis after meeting our eligibility criteria (see Figures 1, 2). The study types, sample sizes, and descriptions of the interventions can be found in Table 1.

**Table 1:** Summary and characteristics of VR studies for the treatment of CRPS (BPD, Body Perception Disturbances; PNI, peripheral nerve injury; HEVR, heartbeat enhanced virtual reality; SD, standard deviation).

**References**	**Title**	**Study design**	**Sample size total (group)/participants**	**Study description**	**Duration of the intervention**	**Main outcome measures**	**Type of VR**
Lewis et al. (32)	Visual illusions modulate body perception disturbance and pain in Complex Regional Pain Syndrome: A randomized trial	Blinded randomized controlled trial	45 patients with CRPS fulfilling the Budapest clinical diagnostic criteria (23 in the experimental group and 22 in the control group)	Participants with refractory upper-limb CRPS and BPD viewed a digital image of their affected hand for one minute. In the therapy group, the image was digitally altered according to the patient's description of how they desired their hand to look. The image remained unaltered in the control group. BPD and pain were measured pre-and post-intervention. A subgroup (21 of the experimental group and 18 controls) was followed up 2 weeks after a course of repeated interventions.	4 weekly intervention sessions	Bath body perception disturbance (BPD) scale Pain intensity numerical rating scale (NRS) Perceptual statement ratings	Mediated VR-system “MIRAGE”
Chau et al. (12)	Immersive virtual reality for pain relief in upper limb complex regional pain syndrome: a pilot study	Pilot study	8 patients with CRPS	An immersive virtual 3D interactive kitchen environment was designed that allowed visualization and manipulation of objects with virtual hands. Participants performed tasks of daily activities as well as guided visualization exercises for a total of 10 sessions. The system permitted the user to freely walk in the premeasured space to explore the environment. Each session lasted ~45 min to an hour. Pain intensity was assessed before and after interventions.	10 sessions of 45–60 min duration	Short-Form McGill Pain Questionnaire (SF-MPQ) Visual Analog Scale (VAS) Wong-Baker FACES (WBF) rating scale Subjective comments of function and symptoms	Immersive VR with head-mounted display
Sato et al. (33)	Nonimmersive virtual reality mirror visual feedback therapy and its application for the treatment of complex regional pain syndrome: an open-label pilot study	Open label pilot study (case series)	5 patients with CRPS fulfilling diagnostic criteria for CRPS of the International Association for the Study of Pain	CRPS patients wore a digital glove with sensors on their non-affected side. A position sensor was attached to the affected side. In several movement tasks, the virtual forearm moved in the same way as the affected side, but the hand and finger movements of the virtual arm were manipulated by the unaffected side.	5–8 sessions weekly intervention sessions	Visual Analog Scale (VAS)	Non-immersive mirror visual feedback Desktop-VR (3D environment presented on a 2D monitor)
Solcà et al. (34)	Heartbeat enhanced immersive virtual reality to treat complex regional pain syndrome	Crossover double-blind study	48 (24 patients with CRPS and 24 age- and sex-matched controls) Patients fulfilled the Harden CRPS criteria for research	Participants were shown a virtual depiction of their affected limb which was flashing in either synchrony or asynchrony (control condition) with their own heartbeat [heartbeat enhanced virtual reality (HEVR)]. The	Two conditions, each repeated three times in succession, with a stimulus duration of 90 s	Subjective pain ratings/VAS force strength heart rate variability (HRV)	Immersive VR combined with biofeedback
				synchronous and asynchronous conditions were repeated three times. Pain and grip strength were assessed.			
Matamala-Gomez et al. (35)	Decreasing pain ratings in chronic arm pain through changing a virtual body: different strategies for different pain types	Clinical trial	19 (9 CRPS type I and 10 with PNI) CRPS type I had been previously diagnosed based on the Budapest criteria for CRPS	Participants were shown a virtual arm at four different transparency sizes and three different sizes. This happened in a single session that lasted 55 min.	Participants completed a single session of 55 min, consisting of four parts: baseline, first test, period of pause, and second test	Pain Intensity-Numeric Rating Scale (PI-NRS) seven-item, seven-point Likert-type virtual reality questionnaire	Immersive VR using head mounted display
Jeon et al. (36)	Application of virtual body swapping to patients with complex regional pain syndrome: a pilot study	Randomized controlled trial	10 patients with CRPS type 1 fulfilling the criteria according to the International Association for the Study of Pain	CRPS patients were randomly assigned to the treatment or control group. All participants were asked to watch the virtual body swapping training video clip with a head-mounted display. The video had a length of 3 min and was filmed from the first person perspective in order for the participant to feel as if they were observing their own body. The video depited 4 ergonomically natural movements consisting of making fists, bending elbows, bending ankles and bending legs. The treatment group was additionally asked to assume a posture similar to the body on the screen and rehearse the movements mentally as if the body presented on the screen was theirs. Pain intensity and BPD was evaluated.	A 3.20 min video clip was played twice with a 1 min pause, the whole training session lasted <10 min	Pain intensity (11-point Likert scale) Body Perception Disturbance Questionnaire (BPDQ), modified single item regarding virtual body swapping illusion (7-point Likert scale)	Immersive VR using Head—Mounted display, recorded 2D—videos
Won et al. (16)	Assessing the feasibility of an open-source virtual reality mirror visual feedback module for complex regional pain syndrome: pilot usability study	Pilot study	9 patients with CRPS	CRPS patients performed a VR-based mirror therapy at least 4 times. The sessions took place once a week. The movement of the patient's uninjured hand was transformed over the midline to animate an avatar hand on the injured side. The task was to bring both the injured and uninjured hands together until they made contact. Pain, physical activity, mood and quality of sleep were assessed.	4–5 training sessions once a week	Pain survey modified from the Brief Pain Inventory Patient reported outcome measures Movement data (head and uninjured hand)	Immersive VR using head-mounted display

### 3.1. Pain reduction

All included studies reported pain levels before and after the respective interventions.

Lewis et al. (32) found a significant reduction in pain intensity after a single exposure compared to controls in a randomized trial study [effect size (ES) = 0.5]. The subgroup of 21 patients evaluated 2 weeks later after repeated intervention showed a sustained significant reduction in pain intensity (ES = 0.7).

In a crossover double-blind study by Solcà et al. (34), patients were shown a virtual depiction of the arm which was flashing either synchronous or asynchronous to their heartbeat. The authors reported a significant reduction in pain ratings after synchronous stimulation. In contrast, asynchronous stimulation did not show a significant effect on pain.

In the study by Matamala-Gomez et al. (35) patients with CRPS and peripheral nerve injury (PNI) were shown a virtual arm at four transparency levels and three sizes. Overall, the VR sessions reduced pain ratings by ~50 %. However, the various presentations of the virtual arm differed regarding their impact on the two patient groups: Increasing the transparency reduced pain levels in CRPS but not in PNI patients while increasing the size worsened pain only in CRPS.

Chau et al. (12) describe that patients frequently presented lower pain scale scores after each of ten VR sessions, but no overall improvement of pain was found after completion of all the sessions. Even though the pain scores did not show a significant change, subjective feedback of the patients included several reports of sustained pain relief.

In the study by Sato et al. (33), four of the five included patients showed over 50% reduction of pain intensity and the mean VAS score decreased from 64 (±14 standard deviation) to 31 (±26) after the sessions.

Jeon et al. (36) did not find any significant changes in pain intensity between the treatment and the control group.

Finally, for Won et al. (16), several participants commented on subjective pain reduction but there were no statistically significant differences regarding pain intensity before and after the interventions.

### 3.2. Body perception disturbances

Two studies (Lewis et al. and Jeon et al.) assessed BPD systematically. Lewis et al. used both the Bath BPD scale and perceptual statement ratings to evaluate BPD. Their single exposure therapy group showed a significantly greater reduction in the Bath BPD scale than the control group (ES = 0.6) (32). Additionally, the liking of the affected hand increased, the sense of heaviness reduced and the sense of lightness increased. There was no difference amongst the groups regarding ownership and sensation. However, the group that was presented with a repeated exposure therapy showed no significant improvement regarding both the Bath BPD scale and perceptual ratings.

Jeon et al. also used the Bath BPD scale and found that the treatment group reported significantly less BPD after treatment than the control group (36).

### 3.3. Limb movement and daily function improvement

Only one study (Solcà et al.) provided systematic data regarding the function of the affected limb. One study (Won et al.) assessed general physical activity. Three studies (Won et al., Sato et al., Chau et al.) report subjective descriptions of individual patients.

Solcà et al. report that grip strength increased after synchronous stimulation while no change was observed after asynchronous stimulation (34).

In the study by Won et al., patients reported subjective pain relief and subjective improvement in daily function in questionnaires while general physical activity remained unaltered (16).

Sato et al. reported distal limb improvement in a patient who improved her wrist and finger range of motion (33).

In the study by Chau et al., one patient stated almost complete resolution of chronic symptoms with increased functional use of the affected limb (12).

## 4. Discussion

To the best of our knowledge, this review is the first to gather the existing information on VR and its use on CRPS.

CRPS has substantial impacts on patients' quality of life and on their physical and psychological well-being (2), thus optimized treatment is crucial. Currently, the exact pathophysiological mechanisms of CRPS are unknown, making it impossible to define a gold-standard therapy for these patients (2). For this reason, many therapeutic interventions are being attempted, including the possible use of VR modalities (37).

VR has been shown to be a potentially reliable and effective treatment option for many chronic pain conditions (38). In this study, we gathered the evidence of the existing studies that have been conducted for VR and CRPS and were able to find many positive outcomes in these patients. We focused our research on evaluating the improvement of pain levels, BPD, and daily function/limb movement. The seven articles used for this revision provided evidence from clinical trials, pilot studies, randomized controlled trials, and double-blinded studies. Although most had a small number of patients included, significant results were found.

From our first objective (reduction in pain levels), significant and subjective outcomes from patients were found. Lewis et al. and Solcà et al. presented statistically significant data incorporating their different VR modalities of desired hand image and HEVR (32, 34). Matamala-Gomez et al. described that varying transparency gives the most significant decrease in pain ratings. Sato et al. interpreted their data indicating that 4/5 participants of their study presented more than 50% in pain reduction from their VR mirror visual feedback therapy. However, as there was no control group, it is unclear to what extent this reduction in pain is due to the intervention and to what extent it is influenced by other factors, such as time. The other studies showed subjective improvement in many patients, but no statistically significant data was found.

Regarding BPD disturbances, Lewis et al. found significant improvement in BPD from a single session of exposure therapy, and Jeon et al. found significantly improved BPD in their virtual body swapping intervention (32, 34).

Ownership illusions were investigated by Solcà et al. and Matamala-Gomez et al. Solcà et al. reported that the synchronous stimulation positively affected ownership (34). Matamala-Gomez et al. found similar results when their participants had higher ownership levels following the intervention (35).

The interventions that allowed to reflect on limb movement/daily function were few and did not have the amount of information that would have been ideal for this review. Jeon et al. were the only ones to statistically analyze limb movement and found that grip strength had improved significantly. Besides this, the other results were completely subjective from patients considering improvements in their limb movement and daily function post-intervention.

The studies all included different modalities of VR, including body-swapping, VR mirror visual feedback, heartbeat-enhanced virtual reality (HEVR), immersive virtual reality and VR limb modulation. These different modalities have important characteristics that can help reflect what VR characteristics may be the best to incorporate into CRPS management. Important observations can be made mainly focusing on pain levels and BPD.

The various VR modalities can be applied to address the different biopsychosocial dimensions of pain. Due to its inherent complexity, VR has the potential to achieve goals such as distraction, modification of behavioral factors, experience of positive events and training of specific movements simultaneously (26).

Hoffman at al. were able to show that the pain-relieving effect of virtual worlds correlates with greater presence. Healthy participants wore VR helmets with displays of different quality and received standardized pain stimuli. The more realistic the graphical representation of the virtual world was, the stronger the reduction in perceived pain was (39). Furthermore, it has been shown that the illusion of embodiment enhances the emotional processing of the virtual environment (27).

Two of the studies included in this review specifically examined embodiment. Solcà et al. evaluated the embodiment perceptions of their patients using a questionnaire (34). Their results showed that HEVR induced a positive feeling of ownership toward the virtual hand during both synchronous and asynchronous stimulation. This effect was greater for synchronous than for asynchronous stimulation, but the difference was not statistically significant.

Matamala-Gomez et al. evaluated the participants' ownership toward the affected limb and found that all the patients except for one had a distorted body image representation (35). Following the visuotactile stimulation of the virtual hand, both the CRPS and the PNI group reported high levels of ownership of the virtual hand. The authors also described that both groups of patients with chronic pain reported significantly higher illusory agency, referring to the illusion that one could control the movements of the hand at will.

Wong et al. conclude that immersive VR interventions have a higher potential than non-immersive applications to overcome the sensation of pain due to the higher input of sensory information (40).

The method introduced by Solcà et al. also incorporates a new concept for pain modulation. They not only use immersive VR but add a cardiac signal monitoring technology. Pain signals and cardiac signals travel in anatomically close paths and are relayed via several common central nervous system regions. Studies have also shown relations between pain-brain and heart-related processes, including physiological pain measures (flexion reflex or pain evoked potentials) that can be reduced during the systolic phase of the cardiac cycle (41, 42). As a bonus, this VR method does not require tactile stimulation, which can often cause allodynia. The authors argue that the synchrony of heartbeat induced pain reduction and that more frequent and more prolonged treatment doses would be possible without any patient discomfort.

A theory for the effect of VR on BPD is explained by Lewis et al. who suggest participants were able to access their innate body schema that had been altered by their pain condition, from viewing a desired appearance of the painful hand. In other words, they hypothesize an incongruence between body image and body scheme that when controlled, can improve BPD and help to alleviate pain. This supports a past study stating that pain can be modulated by targeting BPD (43). Other techniques used, such as virtual body swapping by Jeon et al. also positively impacted BPD. These modalities of VR are consistent with the widely used MVF and GMI therapies. These therapies are driven by the aim to restore cortical reorganization of areas that through maladaptive neuroplasticity changes can be involved in the long-term maintenance of pain and BPD in CRPS. Incorporating these therapies into VR solves many drawbacks of conventional mirror feedback, such as active patient participation, limited range of stimuli, and poor coordination of stimulation parameters since it is not programmable (44, 45). Sato et al. are the first to implement VR to MVF, arguing that this combination is excellent for immersion, engagement, and reward and can motivate patients to complete the task training continuously.

Overall, all VR modalities will activate various brain areas, some of which overlap with areas where adaptive changes have been shown to occur in patients with CRPS (46, 47). Neuroimaging findings are essential for characterizing these areas and their changes in CRPS. In 2014, Pleger et al. found that the gray matter of CRPS patients in the primary motor cortex (M1) was increased contralateral to the CRPS-affected limb, which was inversely related to decreased white matter density of the internal capsule within the ipsilateral brain hemisphere (48).

A later neuroimaging study stated that the medial prefrontal cortex had increased connectivity to the insula in proportion to the intensity of the pain (49). In 2015, a study with advanced neuroimaging fMRI techniques presented evidence that the primary somatosensory cortex (S1) representation was smaller for the affected hand than for the healthy hand of CRPS patients, as predicted. However, they also showed that the S1 representation of the affected hand was no different from that of either hand in controls. Not only this, but the S1 representation of the healthy hand of patients was larger than that of controls' hands. This is striking because it provides an insight toward a hypothesis that CRPS may be associated with an enlarged representation of the healthy hand, not a smaller representation of the affected hand as thought before (28). Additionally, in 2019 Diers et al. confirm S1 alterations by stating that in the S1, the affected hand tends to be smaller than the unaffected hand. The recompiling of evidence on neuroimaging studies for CRPS is valuable for the review since many studies found improvement in pain levels and BPD from methods that stimulate all the brain areas established to be affected by CRPS (50).

In further studies on the application of VR-based therapies in CRPS, factors such as treatment initiation, frequency, intensity, and duration of therapy should be placed on a solid clinical data basis. Furthermore, it would be interesting to find out whether it is possible to predict the response to one of the described therapies in selected patient groups or in which stages of the disease which VR-based therapy is particularly effective. The mechanisms of action of the different VR entities should be better understood and individualized, adaptable therapy concepts should be developed.

While in other diseases a relevant limitation of current VR-based therapies is the lack of realistic haptic experiences and touch by “therapeutic” hands with the corresponding positive effects (27), this can be a decisive advantage especially in CRPS, where local touch is often hardly possible and is experienced as intensifying pain.

This review had various limitations. Firstly, as VR is an emerging concept there is not enough information available to gather a sufficient amount of high-quality evidence for safe inferences about the results. This leaves us with studies providing important conclusions and others with only subjective evidence from the participants. Secondly, the reports primarily available had a very small number of patients. This resulted in authors not being able to make many objective conclusions about their results since the number of participants was not large enough to make statistical analysis, extrapolate their conclusions to larger populations, or compare to other studies. Additionally, there were no longitudinal studies found. Therefore, we have only been able to reflect on a short period after the treatment was applied to the patients. The most amount of time that a patient was formally evaluated after treatment was 2 weeks, but after this timeframe, we do not have any data about the outcome of the patient post-treatment. Finally, the lack of control conditions is an important limitation of the majority of the included studies. Therefore, it cannot be excluded that the results are influenced not only by the interventions but also by factors such as placebo effects, unspecific attention benefits, medication and other treatments.

## 5. Conclusions

The available studies can be interpreted as useful for future studies and to impulse VR technology as a treatment method for CRPS patients, especially in the area of pain management and improvement in BPD. These studies have various limitations, and the pathophysiology of CRPS is still largely unknown. However, its treatment must be attempted from various strategies. VR has shown evidence toward modulating chronic pain disorders, confirmed in many of the studies included in this review. This strategy is valuable in the search for an effective CRPS treatment. The use of VR strategies such as limb modulation, immersive VR, body switching, mirror feedback, and even immersive VR with cardiac signaling have various approaches that are related to the cortical reorganization theories in BPD and chronic pain, pain modulation pathways, and the psychological aspects of body image and body schema. For these reasons, the studies included have logical methods in their VR protocols. The overall subjective and objective results reflect a positive effect of VR on CRPS patients, with most evidence going toward pain reduction and some evidence on BPD improvement. This makes way for future studies with greater cohorts of patients and the inclusion of longer follow-up times to assess the long terms effects of VR on CRPS. Future work on VR-based therapy for CRPS patients should include adequate control groups.

## Author contributions

MA-H, JTB and SCA: clinical and methodological expertise, conception, and writing of the review. CS and MG: development of the search strategy, clinical expertise and advice, and revision of the manuscript. BMH: clinical and methodological expertise, supervision, and revision of the manuscript. EK: clinical and methodological expertise and advice, conception, and writing of the review. All authors contributed to the article and approved the submitted version.

## References

[1] Harden NR Bruehl S Perez RSGM Birklein F Marinus J Maihofner C. Validation of proposed diagnostic criteria (the “Budapest Criteria”) for complex regional pain syndrome. Pain. (2010) 150:268–74. 10.1016/j.pain.2010.04.030 20493633 PMC2914601

[2] Dey S Guthmiller KB Varacallo M. Complex Regional Pain Syndrome. Statpearls. Treasure Island, FL: StatPearls Publishing Copyright © 2021, StatPearls Publishing LLC (2021). 28613470

[3] Bussa M Guttilla D Lucia M Mascaro A Rinaldi S. Complex regional pain syndrome type I: a comprehensive review. Acta Anaesthesiol Scand. (2015) 59:685–97. 10.1111/aas.12489 25903457

[4] Goebel A. Complex regional pain syndrome in adults. Rheumatology. (2011) 50:1739–50. 10.1093/rheumatology/ker202 21712368

[5] Sandroni P Benrud-Larson LM McClelland RL Low PA. Complex regional pain syndrome type I: incidence and prevalence in olmsted county, a population-based study. Pain. (2003) 103:199–207. 10.1016/S0304-3959(03)00065-4 12749974

[6] Misidou C Papagoras C. Complex regional pain syndrome: an update. Mediterr J Rheumatol. (2019) 30:16–25. 10.31138/mjr.30.1.16 32185338 PMC7045919

[7] Sebastin SJ. Complex regional pain syndrome. Indian J Plast Surg. (2011) 44:298–307. 10.1055/s-0039-1699507 22022040 PMC3193642

[8] Libon DJ Schwartzman RJ Eppig J Wambach D Brahin E Peterlin BL. Neuropsychological deficits associated with complex regional pain syndrome. J Int Neuropsychol Soc. (2010) 16:566–73. 10.1017/S1355617710000214 20298641

[9] Trojan J Diers M Valenzuela-Moguillansky C Torta DME. Body, space, and pain. Front Hum Neurosci. (2014) 8:369. 10.3389/fnhum.2014.00369 24904392 PMC4035599

[10] Kuttikat A Noreika V Shenker N Chennu S Bekinschtein T Brown CA. Neurocognitive and neuroplastic mechanisms of novel clinical signs in Crps. Front Hum Neurosci. (2016) 10:16. 10.3389/fnhum.2016.00016 26858626 PMC4728301

[11] Mölbert SC Klein L Thaler A Mohler BJ Brozzo C Martus P. Depictive and metric body size estimation in anorexia nervosa and bulimia nervosa: a systematic review and meta-analysis. Clin Psychol Rev. (2017) 57:21–31. 10.1016/j.cpr.2017.08.005 28818670

[12] Chau B Phelan I Ta P Chi B Loyola K Yeo E. Immersive virtual reality for pain relief in upper limb complex regional pain syndrome: a pilot study. Innov Clin Neurosci. (2020) 17:47–52. 32802594 PMC7413329

[13] Storz C Kraft E. [Occupational therapy for complex regional pain syndrome]. Schmerz. (2021) 35:285–93. 10.1007/s00482-021-00559-0 34137926

[14] Kraft E Storz C Ranker A. [Physical therapy in the treatment of complex regional pain syndrome]. Schmerz. (2021) 35:363–72. 10.1007/s00482-021-00577-y 34529155

[15] Kim IS Hyun SE Park J Lim JY. Understanding the rehabilitation needs of korean patients with complex regional pain syndrome. Ann Rehabil Med. (2020) 44:218–27. 10.5535/arm.19084 32475093 PMC7349043

[16] Won AS Barreau AC Gaertner M Stone T Zhu J Wang CY. Assessing the feasibility of an open-source virtual reality mirror visual feedback module for complex regional pain syndrome: pilot usability study. J Med Internet Res. (2021) 23:e16536. 10.2196/16536 34037530 PMC8190641

[17] Pourmand A Davis S Marchak A Whiteside T Sikka N. Virtual reality as a clinical tool for pain management. Curr Pain Headache Rep. (2018) 22:53. 10.1007/s11916-018-0708-2 29904806

[18] Hao J Xie H Harp K Chen Z Siu KC. Effects of virtual reality intervention on neural plasticity in stroke rehabilitation: a systematic review. Arch Phys Med Rehabil. (2022) 103:523–41. 10.1016/j.apmr.2021.06.024 34352269

[19] Calafiore D Invernizzi M Ammendolia A Marotta N Fortunato F Paolucci T. Efficacy of virtual reality and exergaming in improving balance in patients with multiple sclerosis: a systematic review and meta-analysis. Front Neurol. (2021) 12:773459. 10.3389/fneur.2021.773459 34956054 PMC8702427

[20] Chuan A Zhou JJ Hou RM Stevens CJ Bogdanovych A. Virtual reality for acute and chronic pain management in adult patients: a narrative review. Anaesthesia. (2021) 76:695–704. 10.1111/anae.15202 32720308

[21] Mütterlein J. The three pillars of virtual reality? Investigating the role of immersion, presence, and interactivity. In: *51st Hawaii International Conference on System Sciences*. Big Island, Hawaii. (2018). 10.24251/HICSS.2018.174

[22] Kim G Biocca F. Immersion in virtual reality can increase exercise motivation and physical performance. In: International Conference on Virtual, Augmented and Mixed Reality. Cham: Springer (2018). p. 94–102.

[23] Slater M. Immersion and the illusion of presence in virtual reality. Br J Psychol. (2018) 109:431–3. 10.1111/bjop.12305 29781508

[24] Slater M Sanchez-Vives MV. Enhancing our lives with immersive virtual reality. Front Robot AI. (2016) 3:74. 10.3389/frobt.2016.00074

[25] Kilteni K Groten R Slater M. The sense of embodiment in virtual reality. Presence. (2012) 21:373–87. 10.1162/PRES_a_00124

[26] Trost Z France C Anam M Shum C. Virtual reality approaches to pain: toward a state of the science. Pain. (2021) 162:325–31. 10.1097/j.pain.0000000000002060 32868750

[27] Donegan T Ryan BE Sanchez-Vives MV Swidrak J. Altered bodily perceptions in chronic neuropathic pain conditions and implications for treatment using immersive virtual reality. Front Hum Neurosci. (2022) 16:1024910. 10.3389/fnhum.2022.1024910 36466621 PMC9714822

[28] Di Pietro F Stanton TR Moseley GL Lotze M McAuley JH. Interhemispheric somatosensory differences in chronic pain reflect abnormality of the healthy side. Hum Brain Mapp. (2015) 36:508–18. 10.1002/hbm.22643 25256887 PMC6869612

[29] Filbrich L Alamia A Verfaille C Berquin A Barbier O Libouton X. Biased visuospatial perception in complex regional pain syndrome. Sci Rep. (2017) 7:9712. 10.1038/s41598-017-10077-8 28852115 PMC5574889

[30] Alemanno F Houdayer E Emedoli D Locatelli M Mortini P Mandelli C. Efficacy of virtual reality to reduce chronic low back pain: proof-of-concept of a non-pharmacological approach on pain, quality of life, neuropsychological and functional outcome. PLoS ONE. (2019) 14:e0216858. 10.1371/journal.pone.0216858 31120892 PMC6532874

[31] Munn Z Peters MDJ Stern C Tufanaru C McArthur A Aromataris E. Systematic review or scoping review? Guidance for authors when choosing between a systematic or scoping review approach. BMC Med Res Methodol. (2018) 18:143. 10.1186/s12874-018-0611-x 30453902 PMC6245623

[32] Lewis JS Newport R Taylor G Smith M McCabe CS. Visual illusions modulate body perception disturbance and pain in complex regional pain syndrome: a randomized trial. Eur J Pain. (2021) 25:1551–63. 10.1002/ejp.1766 33759278

[33] Sato K Fukumori S Matsusaki T Maruo T Ishikawa S Nishie H. Nonimmersive virtual reality mirror visual feedback therapy and its application for the treatment of complex regional pain syndrome: an open-label pilot study. Pain medicine. (2010) 11:622–9. 10.1111/j.1526-4637.2010.00819.x 20202141

[34] Solcà M Ronchi R Bello-Ruiz J Schmidlin T Herbelin B Luthi F. Heartbeat-enhanced immersive virtual reality to treat complex regional pain syndrome. Neurology. (2018) 91:e479–e89. 10.1212/WNL.0000000000005905 29980635

[35] Matamala-Gomez M Diaz Gonzalez AM Slater M Sanchez-Vives MV. Decreasing pain ratings in chronic arm pain through changing a virtual body: different strategies for different pain types. J Pain. (2019) 20:685–97. 10.1016/j.jpain.2018.12.001 30562584

[36] Jeon B Cho S Lee JH. Application of virtual body swapping to patients with complex regional pain syndrome: a pilot study. Cyberpsychol Behav Soc Netw. (2014) 17:366–70. 10.1089/cyber.2014.0046 24892199

[37] Mallari B Spaeth EK Goh H Boyd BS. Virtual reality as an analgesic for acute and chronic pain in adults: a systematic review and meta-analysis. J Pain Res. (2019) 12:2053–85. 10.2147/JPR.S200498 31308733 PMC6613199

[38] Ahmadpour N Randall H Choksi H Gao A Vaughan C Poronnik P. Virtual reality interventions for acute and chronic pain management. Int J Biochem Cell Biol. (2019) 114:105568. 10.1016/j.biocel.2019.105568 31306747

[39] Hoffman HG Seibel EJ Richards TL Furness TA Patterson DR Sharar SR. Virtual reality helmet display quality influences the magnitude of virtual reality analgesia. J Pain. (2006) 7:843–50. 10.1016/j.jpain.2006.04.006 17074626

[40] Wong KP Tse MMY Qin J. Effectiveness of virtual reality-based interventions for managing chronic pain on pain reduction, anxiety, depression and mood: a systematic review. Healthcare. (2022) 10. 10.3390/healthcare10102047 36292493 PMC9602273

[41] Park H-D Correia S Ducorps A Tallon-Baudry C. Spontaneous fluctuations in neural responses to heartbeats predict visual detection. Nat Neurosci. (2014) 17:612–8. 10.1038/nn.3671 24609466

[42] Bruehl S Chung OY. Interactions between the cardiovascular and pain regulatory systems: an updated review of mechanisms and possible alterations in chronic pain. Neurosci Biobehav Rev. (2004) 28:395–414. 10.1016/j.neubiorev.2004.06.004 15341037

[43] Lewis JS Schweinhardt P. Perceptions of the painful body: the relationship between body perception disturbance, pain and tactile discrimination in complex regional pain syndrome. Eur J Pain. (2012) 16:1320–30. 10.1002/j.1532-2149.2012.00120.x 22407949

[44] Méndez-Rebolledo G Gatica-Rojas V Torres-Cueco R Albornoz-Verdugo M Guzmán-Muñoz E. Update on the effects of graded motor imagery and mirror therapy on complex regional pain syndrome type 1: a systematic review. J Back Musculoskelet Rehabil. (2017) 30:441–9. 10.3233/BMR-150500 27858687

[45] Rjosk V Kaminski E Hoff M Sehm B Steele CJ Villringer A. Mirror visual feedback-induced performance improvement and the influence of hand dominance. Front Hum Neurosci. (2016) 9:702. 10.3389/fnhum.2015.00702 26834605 PMC4720001

[46] Maihöfner C Baron R DeCol R Binder A Birklein F Deuschl G. The motor system shows adaptive changes in complex regional pain syndrome. Brain J Neurol. (2007) 130:2671–87. 10.1093/brain/awm131 17575278

[47] Maihöfner C Handwerker HO Neundörfer B Birklein F. Patterns of cortical reorganization in complex regional pain syndrome. Neurology. (2003) 61:1707–15. 10.1212/01.WNL.0000098939.02752.8E 14694034

[48] Pleger B Draganski B Schwenkreis P Lenz M Nicolas V Maier C. Complex regional pain syndrome type i affects brain structure in prefrontal and motor cortex. PLoS ONE. (2014) 9:e85372. 10.1371/journal.pone.0085372 24416397 PMC3887056

[49] Baliki MN Mansour AR Baria AT Apkarian AV. Functional reorganization of the default mode network across chronic pain conditions. PLoS ONE. (2014) 9:e106133. 10.1371/journal.pone.0106133 25180885 PMC4152156

[50] Diers M. Neuroimaging the pain network - implications for treatment. Best Pract Res Clin Rheumatol. (2019) 33:101418. 10.1016/j.berh.2019.05.003 31703795

